# Impact of COVID-19 Pandemic on Consumption of Anxiolytics, Antipsychotics, and Antidepressants in South Italian Region

**DOI:** 10.3390/life15040652

**Published:** 2025-04-16

**Authors:** Vittoria Satriani, Emanuela Santoro, Mario Capunzo, Rosaria Flora Ferrara, Roberta Manente, Biagio Santella, Gianluigi Franci, Francesco De Caro, Giovanni Boccia

**Affiliations:** 1 Department of Medicine, Surgery and Dentistry “Scuola Medica Salernitana”, University of Salerno, 84081 Salerno, Italy; vsatriani@unisa.it (V.S.); mcapunzo@unisa.it (M.C.); rosferrara@unisa.it (R.F.F.); bsantella@unisa.it (B.S.); gfranci@unisa.it (G.F.); fdecaro@unisa.it (F.D.C.); gboccia@unisa.it (G.B.); 2 DAI Department of Health Hygiene and Evaluative Medicine, A.O.U. San Giovanni di Dio e Ruggi d’Aragona, 84131 Salerno, Italy; 3 Clinical Pathology and Microbiology Unit, A.O.U. San Giovanni di Dio e Ruggi d’Aragona, 84131 Salerno, Italy; manente392@gmail.com; 4 UOS Microbiology and Virology, A.O.U. San Giovanni di Dio e Ruggi d’Aragona, 84131 Salerno, Italy; 5 Public Health Laboratory for the Analysis of Community Health Needs, Department of Medicine and Surgery, University of Salerno, Baronissi Campus, 84081 Baronissi, Italy; 6 A.O.U. San Giovanni di Dio e Ruggi d’Aragona, 84081 Salerno, Italy; 7 U.O.C. Hospital and Epidemiological Hygiene, A.O.U. San Giovanni di Dio e Ruggi d’Aragona, 84131 Salerno, Italy

**Keywords:** COVID-19 pandemic, psychotropic medication, patients, anxiolytics, antipsychotics, antidepressants, public health, Italy

## Abstract

The COVID-19 lockdown had significant consequences on the mental health of millions of people, leading to the increasing prescription and use of psychotropic drugs. Due to the lack of data in the current literature, this study aims to estimate the trends in the consumption of anxiolytics, antipsychotics, and antidepressants before and during the pandemic on people in the South Italian region. We conducted a retrospective observational study, retrieving prescriptions of anxiolytics, antipsychotics, and antidepressants dispensed in pharmacies of the Basilicata region (South Italy) for the period 2019–2021. We presented the data, expressed in Daily Defined Doses (DDDs) for 1000 persons/day (DHD), on a total resident population of 95,021, dividing the age groups into categories <30, 30–50, 50–70, and >70 years. We conducted a linear regression model to examinate consumption trends across years. Software XLSTAT was used for statistical analysis. During the study period, more than 85,000 boxes of psychotropic medications were dispensed. The research showed an increase in the sales of all three categories of drugs examined, with a greater rise in benzodiazepines (88.5%) and selective serotonin reuptake inhibitors (81.9%) in 2021 compared to 2019, especially among women under 30 years. The COVID-19 pandemic had led to an increase in the consumption of psychotropic drugs, confirming the significant impact on the population’s mental health.

## 1. Introduction

COVID-19 emerged in late 2019 in Wuhan, China, with cases first reported on 31 December 2019, leading to the identification of the SARS-CoV-2 virus as the cause of acute respiratory syndrome by 20 January 2020 [1,2].

On 11 March 2020, the World Health Organization (WHO) officially declared the onset of the pandemic, alarmed by the levels of spread and severity of the virus [3].

Italy was the second country after China to be severely affected at the start of the pandemic. As of 15 February 2022, 12.1 million confirmed cases and 151,000 deaths had been recorded nationally [4].

On 31 March 2022, a state of emergency was declared in Italy, according to DL 24/2022; on 5 May 2023, the WHO declared the end of the health emergency while emphasizing the need to continue monitoring the progression of the virus [5].

The COVID-19 pandemic had a significant impact on public health, influencing the way hospital and home-based conditions were managed and especially on mental health [6]. Mandatory lockdowns have undoubtedly changed people’s daily routines and external stimuli have inevitably decreased. Smart working or even job loss in some cases, social isolation, and loneliness have negatively affected mental health. Many people also faced the challenge of ensuring access to basic necessities or had to take on the responsibility of caring for family members severely affected by the virus, which could also contribute to increased stress level [7].

Those most exposed to the risk of developing mental health problems are women, young people, those suffering from sleep disorders, and those already in fragile health [8]. The “Istituto Superiore di Sanità” (ISS) conducted the first study in Italy on the prevalence of depressive symptoms during 2020 and compared the results obtained with those detected in the previous two years. During the phases of the lockdown related to the COVID-19 pandemic, there was an increase in depressive symptoms among Italians with a significantly higher impact among young people aged between 18 and 34 [9].

Studies conducted in China have shown that women have greater risk factors that intensify during chronic environmental stress, such as a pandemic, that exposes them to anxiety and depression more than men and, based on understanding the levels of psychological impact during the initial phase of the COVID-19 epidemic through the Impact of Event Scale-Revised (IES-R) and the Depression, Anxiety and Stress Scale (DASS-21), highlighted that the female gender was significantly associated with a greater psychological impact of the outbreak and higher levels of stress, anxiety, and depression [10,11].

The consumption of psychotropic drugs during the pandemic era represented a significant health emergency for two reasons [12]. The first relates to the difficulties in managing patients already suffering from psychiatric disorders, whose symptoms worsened due to the new stress conditions [13]. This happened due the changed living conditions which made the management of clinical pictures complicated, such as those of schizophrenic subjects. Research has highlighted how veterans with multimorbidity reported that the correlation between physical and emotional symptoms made it more difficult to manage their health and daily activities [14]. Furthermore, factors that could exacerbate the condition of patients with psychosis or depression were certainly their inability to self-care, neurocognitive impairment, and compromised immune function [7,15].

The second concerns the increased consumption of psychotropic drugs among the general population. Many people, particularly those who had never had prior psychiatric disorders, sought relief through medication to cope with anxiety, insomnia, depression, and stress. Studies conducted in Europe and the United States about the use of antidepressants provide compelling evidence of an increase in antidepressant prescriptions during the pandemic compared to the pre-pandemic period [16]. The greater availability of medications, combined with the general and unexpected psychosis of patients, associated with the difficulty of accessing medical consultations and inadequate healthcare human resources contributed to a wider and, in some cases, improper use of psychotropic drugs [17].

The pandemic highlighted not only the psychological vulnerability of a significant part of the population but also the gaps in psychiatric healthcare systems due to the combination of increased demand, disruption in services, and pre-existing disparities.

In Italy, 13% of territorial centers remained closed, and 25% reduced their opening hours; hospital psychiatric consultations (−25%), individual psychotherapy (−65%), and group therapy (−90-95%) experienced a significant decrease [18].

The reasons can be found in the reduction in access to In-Person Care as many mental health facilities transitioned to virtual care, the staff overwhelm (such as burnout) that led to a longer wait list for appointments and less frequent follow-ups, the disruption of ongoing therapy as clinics closed or reduced services and, finally, the lack of crisis intervention resources because in many areas, crisis intervention services were underfunded or unavailable to help patients during acute mental crises.

The analysis was conducted on pharmacological therapies involving the use of anxiolytics, antipsychotics, and antidepressants dispensed by public pharmacies and prescribed by general practitioners to patients in Italy. The study was conducted in the Basilicata region, a small region in southern Italy, due to the lack of literature, as previous studies have been conducted in more populous regions in northern Italy such as Lombardy [19]. We collected sales data from pharmacies in the city of Potenza, the regional capital, and in the municipalities of Avigliano, Barile, Maratea, and Lauria, with the aim of providing an overview on how the consumption of anxiolytics, antipsychotics, and antidepressants has changed and how the COVID-19 impacted on the mental health of the population studied.

The purpose of this study is to find real-world evidence for the hypotheses already reported in the literature about the correlation between the COVID-19 pandemic and mental health, showing that there has indeed been an increase in prescriptions and consumption of psychotropic drugs in response to the emotional vulnerability caused by the pandemic.

## 2. Materials and Methods

We analyzed the consumption of medications based on the total number of units sold (boxes) from the information flow of pharmaceutical prescriptions covered by the National Health Service (SSN in Italy). The healthcare databases available in the individual pharmacies selected for the study collect patient data on all medicines reimbursed by the National Healthcare Service (NHS) and dispensed by local community pharmacies, thanks to the Italian Health Insurance Card. Patients with at least one prescription/dispensing of a psychotropic medication during the study period were considered. The data were collected in full compliance with privacy regulations under Article 13 of European Regulation 2016/679, as, thanks to the health insurance card system, it is possible to retrieve information about each patient’s year of birth, and thus their age and gender. The data were extracted from 1 January 2019, the year prior to the pandemic, and updated to 31 December 2021.

### 2.1. Study Design and Data Source

The data collected are based on prescriptions dispensed through local pharmacies located in the Basilicata region. This region, located in southern Italy, according to the DEMO ISTAT data updated to 1 January 2022, was the reference period for our study and has a total population of 541,168. It is divided into two provinces: Potenza with 100 municipalities and Matera with 31 municipalities. The province of Potenza was examined because it is the most populous and largest, with a population of 349,616, with a prevalence of female gender (50.7%) and a predominant age group of 55 to 64 years (16.2%) [20]. In addition to extracting data from pharmacies in the city of Potenza, the regional capital and largest city in Basilicata, more peripheral areas were also considered due to the lack of data in the literature on the subject, particularly regarding the consumption of psychotropic drugs in rural areas. Specifically, the municipalities of Avigliano, Barile, Lauria, and Maratea were examined. These municipalities were selected as they are the most representative in terms of population density. The selection included areas with varying numbers of inhabitants, geographic locations, and employment patterns, to ensure a broad and diverse sample, making our study as realistic as possible.

According to the DEMO ISTAT data updated on 1 January 2022, the municipality of Potenza, Basilicata’s capital region, has a population of 64,850 divided into 31,168 men (%) and 33,682 women (52%); the municipality of Avigliano, a city primarily focused on commerce, has a population of 10,716 divided into 5223 men (49%) and 5493 women (51%); the municipality of Barile, an area with an industrial vocation, has a population of 2621 divided into 1284 men (49%) and 1337 women (51%); the municipality of Lauria, an inland area with an agricultural vocation, has a population of 12,017, divided into 5821 men (48%) and 6196 women (52%); the municipality of Maratea, a city with a primarily touristic vocation, has a population of 4817, divided into 2369 men (49%) and 2448 women (51%) [20].

Pharmacies were selected using simple random sampling. For the city of Potenza, data were extracted from 9 pharmacies out of the 21 in the city. For the municipality of Avigliano, data were gathered from 1 pharmacy out of 3. In the municipality of Barile, data were extracted from the only pharmacy available in the area. For the municipality of Lauria, data were taken from 1 pharmacy out of 4, and finally, for the municipality of Maratea, data were collected from 1 pharmacy out of 2.

The drug groups selected for the analysis were the most sold ones and chosen to examine the trends of drug consumption from different pharmacological families with varying pharmacokinetic parameters to evaluate as many pharmaco-therapeutic approaches as possible.

Alprazolam and lorazepam were selected as two benzodiazepines with different pharmacological half-lives (10.12 h for alprazolam and 9.5–20 h for lorazepam). Among the antipsychotics, clozapine and quetiapine were chosen, as they belong to two different families. Clozapine is an atypical antipsychotic, while quetiapine is a typical antipsychotic. Finally, regarding antidepressants, paroxetine and citalopram were selected, both belonging to the SSRI family (the most used antidepressant class due to its manageability). Citalopram is relatively selective for the serotonin receptor, while paroxetine also shows significant sensitivity to the muscarinic acetylcholine receptors M1 and M3. Furthermore, they differ in their half-life duration (35 h for citalopram and 21 h for paroxetine). For each pharmacological category, the classification established by the World Health Organization Collaborating Centre (WHOCC) for Drug Statistics Methodology was also indicated as follows: N05B (anxiolytics), N05A (antipsychotics), N06A (antidepressants). For each patient, gender and age were considered; the categories are under 30, to evaluate the trend of medication consumption in the younger population; 30–50 years, to assess consumption in the adult population, which includes workers and families who, as reported in the literature [21], were greatly affected by the stress of the pandemic; 50–70 years, to assess the trend of medication consumption in “young elderly” individuals who developed anxiety, fear, and depression due to their comorbidities combined with the negative impact of the pandemic [22]; and finally, over 70, to evaluate how the consumption of the three drug categories was managed by older adults.

In addition to the sales’ details, for the percentage calculation on overall population consumption, we presented the data as the number of boxes dispensed considering the annual consumption rate, expressed using the standard Daily Defined Doses (DDDs) for 1000 persons/day (DHD), which estimate how many people in 1000 are receiving a DDD per day.

Standard DDD is the technical unity of measurement recommended by the World Health Organization (WHO) for drug utilization studies and is defined as the assumed average maintenance dose per day for a drug used for its main indication in adults and for a specific route of administration [23]. We used the following formula to estimate DHDs:

$$DHD=\frac{\mathrm{Registered}\;\mathrm{consumption}\;\mathrm{of}\;\mathrm{the}\;\mathrm{active}\;\mathrm{ingredient}\;\times \;1000\;\mathrm{inhabitant}}{\mathrm{Standard}\;\mathrm{DDD}\;\times \;\mathrm{no}.\;\mathrm{inhabitants}/\mathrm{period}\;365\;\mathrm{days}}$$

To calculate this measurement, we considered the registered consumption of the active ingredient as the numerator. This value was derived by multiplying the total number of packages sold by the number of pills per package and milligrams of active ingredient per pill. The result was then multiplied by 1000 inhabitants. In the denominator, we used the standard DDD value, which was then multiplied by the total population and the number of days in a year (365).

The boxes considered for the study contained the following information: 20 pills for lorazepam (2.5 mg × pill), 20 pills for alprazolam (1 mg × pill), 28 pills for clozapine (25 mg × pill), 60 pills for quetiapine, (100 mg × pill), 28 pills for paroxetine (20 mg × pill), and 28 pills for citalopram (20 mg × pill). For Potenza and the five municipalities, the considered populations had 64,850 and 30,171 inhabitants, respectively.

### 2.2. Statistical Analysis

The total number of medicine packages sold was described in a percentage ratio for the year considered and divided by the province and city. The annual percentage variation (Δ%) was calculated as the difference between two values expressed as a percentage for the initial value. The data are presented as absolute frequency and relative frequency in Supplementary Table S1. To assess differences in drug consumption rates over the years, we applied parametric and non-parametric statistical tests, depending on data distribution and normality assumptions. Variables representing gender, age group, year, and consumption rate. Data normalization was checked using the Shapiro–Wilk test to determine whether a parametric or non-parametric approach was more appropriate. A linear regression model was used to examine temporal trends in consumption across years, with year as an independent variable and consumption rate as the dependent variable; for non-normally distributed data, a Spearman correlation test was applied. Differences in drug consumption rates between years (2019 vs. 2020, 2020 vs. 2021, and 2019 vs. 2021) were assessed using paired *t*-tests when the data followed a normal distribution. The Wilcoxon signed-rank test was applied as a non-parametric alternative to compare the distributions. A significance level of α = 0.05 was used for all statistical tests. XLSTAT was used for statistical analysis (Lumivero (2024); XLSTAT statistical and data analysis solution, Paris, France).

## 3. Results

Over the period 2019–2021, an annual average of 26,023 boxes of anxiolytics, 8316 antipsychotics, and 14,649 antidepressants, for a total 48,988 boxes, were dispensed in the city of Potenza. The total psychotropic consumption increased from 2019 to 2021 (Table 1), with a percentage increase in the consumption of anxiolytics from 23.16% in 2019 to 43.67% in 2021, of antipsychotics from 18.27% in 2019 to 47.64% in 2021, and of antidepressants from 23.81% in 2019 to 43.30% in 2021.

An annual average of 15,564 boxes of anxiolytics, 8661 antipsychotics, and 12,169 antidepressants, for a total 36,394 boxes, were dispensed in the municipalities of the province of Potenza. The total psychotropic consumption increased from 2019 to 2021. In the study period, the highest change in consumption occurred for anxiolytics, considering the total number of sales amounting to 26,023 for the city and 15,564 for the municipalities (Table 1), with a percentage increase in the consumption of anxiolytics from 19.12% in 2019 to 47.26% in 2021, of antipsychotics from 18.14% in 2019 to 50.24% in 2021, and of antidepressants from 24.42% in 2019 to 41.10% in 2021.

**Figure 1:**
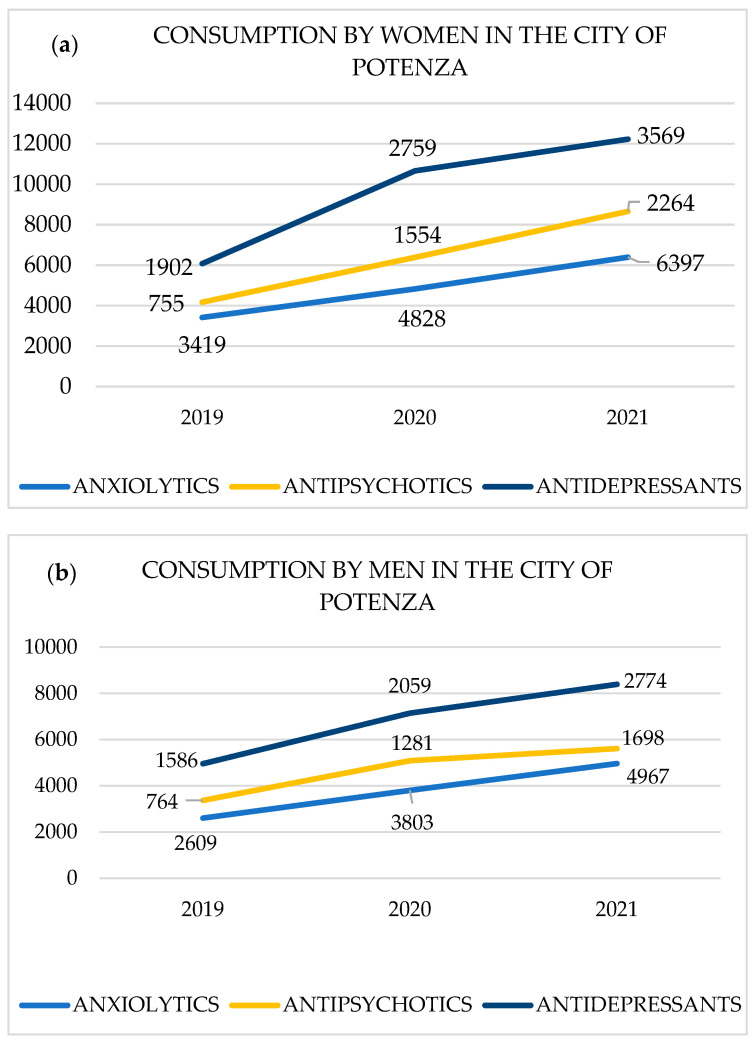
(**a**,**b**) Evolution of pharmacological consumption (number of boxes) by gender for the period 2019–2021 in the city of Potenza.

An upward trend on consumption was observed for the three subgroups of drugs studied and for both sexes, steeper for anxiolytics and antidepressants. In the city of Potenza, the psychotropic most consumed by women were anxiolytics with the number of prescription increased from 3419 in 2019 to 6397 in 2021 with a total of 14,644 boxes sold in 2019–2021, in second place were antidepressants from 1902 in 2019 to 3569 in 2021 with a total of 8230 boxes sold in 2019–2021, and in third place antipsychotics from 755 in 2019 to 2264 in 2021 with a total of 4573 boxes sold in 2019–2021 (Figure 1a).

The psychotropic most consumed by men were anxiolytics with the number of prescription increased from 2609 in 2019 to 4967 in 2021 with a total of 11,379 boxes sold in 2019–2021, in second place were antidepressants from 1586 in 2019 to 2774 in 2021 with a total of 6419 boxes sold in 2019–2021, and in third place were antipsychotics from 764 in 2019 to 1698 in 2021, with a total of 3743 boxes sold in 2019–2021 (Figure 1b).

**Figure 2:**
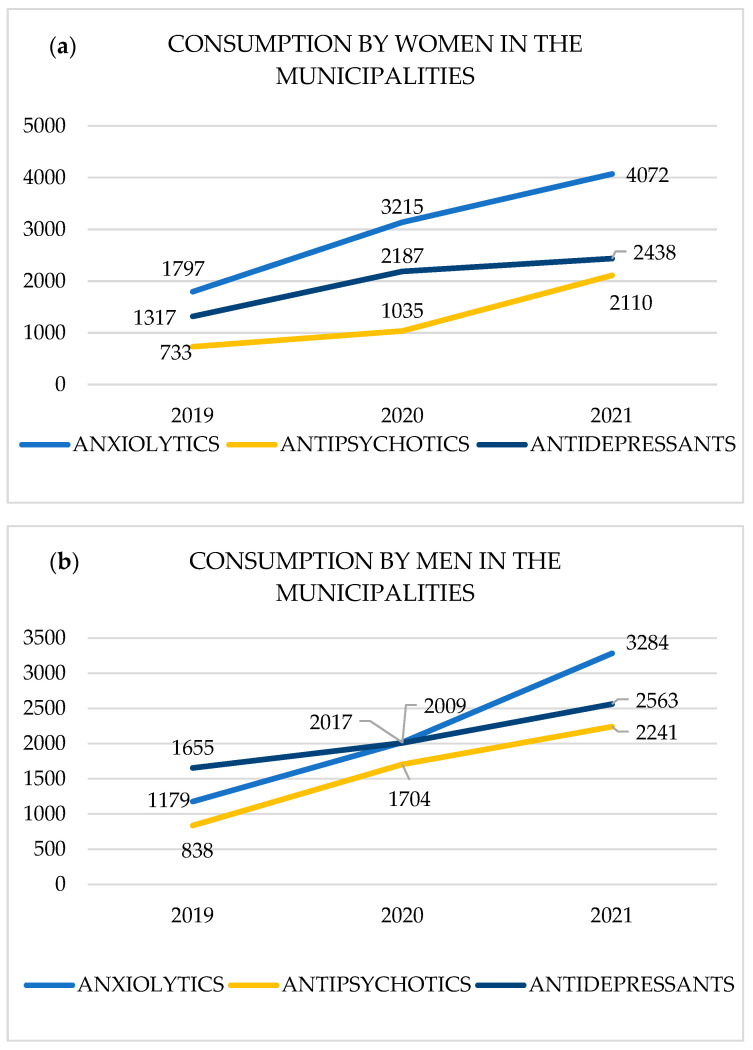
(**a**,**b**) Evolution of pharmacological consumption (number of boxes) by gender for the period 2019–2021 in the municipalities of Potenza (Avigliano, Barile, Lauria, Maratea).

In the municipalities of Potenza province, the psychotropic most consumed by women were anxiolytics with the number of prescriptions increased from 1797 in 2019 to 4072 in 2021 with a total of 9084 boxes sold in 2019–2021, in second place were antidepressants from 1317 in 2019 to 2438 in 2021 with a total of 5942 boxes sold in 2019–2021, and in third place were antipsychotics from 733 in 2019 to 2110 in 2021 with a total of 3878 boxes sold in 2019–2021 (Figure 2a).

The psychotropic most consumed by men were anxiolytics with the number of prescription increased from 1179 in 2019 to 3284 in 2021 with a total of 6480 boxes sold in 2019–2021, in second place were antidepressants from 1655 in 2019 to 2563 in 2021 with a total of 6227 boxes sold in 2019–2021, and in third place were antipsychotics from 838 in 2019 to 2241 in 2021, with a total of 4783 boxes sold in 2019–2021. Finally, it can be noted that in 2019, antidepressants were the most sold pharmaceutical category, while in 2021, the most consumed medications were anxiolytics (Figure 2b).

When analyzing the consumption by sex and age (Table 2, Table 3 and Table 4), a higher consumption increase is found for women compared to men. Overall, for both men and women, the highest consumption are found in younger ages (<30 years) in the city of Potenza and in the older age groups (50–70 or older) in the municipalities of Avigliano, Barile, Maratea, and Lauria. Lorazepam in the city of Potenza has the highest DHD value recorded for men aged 50–70 years in 2021 (1.28 DHD out of a total of 4.43 DHD for the same year). In the municipalities, the highest DHD value is registered by women over 70 years old in 2021 (1.20 DHD out of a total of 6.17 DHD for the same year). Lorazepam shows a significant increase in consumption among men aged 30–50 between 2019 and 2020 (*p* < 0.05). In the city of Potenza, alprazolam has the highest DHD value recorded for women under 30 years in 2021 (1.51 DHD out of a total of 5.17 DHD for the same year). In the municipalities, the highest DHD value is recorded by women aged 50–70 years in 2021 (2.02 DHD out of a total of 7.18 DHD for the same year). Alprazolam shows a significant increase in consumption among men under 30 between 2020 and 2021, as well as among men aged 30–50 between 2019 and 2020 (*p* < 0.05) (Table 2).

**Table 2:** Evolution of DHDs for anxiolytics by age and gender in the period 2019–2021. PZ = city of Potenza. PROV = municipalities of Avigliano, Barile, Lauria, and Maratea in the province of Potenza. DHD = DDDs for 1000 persons/day. F = female. M = male. Highest value for each age group and year is written in bold.

ANXIOLYTICS						
	DHD 2019		DHD 2020		DHD 2021	
LORAZEPAM	PROV	PZ	PROV	PZ	PROV	PZ
**F < 30**	0.15	0.54	0.25	0.63	0.22	0.82
**M < 30**	0.04	0.28	0.18	0.44	0.15	0.69
**F 30–50**	0.04	0.12	0.44	0.20	0.87	0.25
**M 30–50**	0.11	0.13	0.54	0.25	0.86	0.34
**F 50–70**	0.29	0.29	0.72	0.31	0.98	0.57
**M 50–70**	0.27	**0.61**	0.44	**0.90**	0.87	**1.28**
**F > 70**	**0.54**	0.11	**0.91**	0.19	**1.20**	0.24
**M > 70**	0.44	0.08	0.64	0.11	1.03	0.24
**TOTAL**	1.87	2.17	4.12	3.05	6.17	4.43
	**DHD 2019**		**DHD 2020**		**DHD 2021**	
**ALPRAZOLAM**	**PROV**	**PZ**	**PROV**	**PZ**	**PROV**	**PZ**
**F < 30**	0.17	**0.85**	0.24	**1.24**	0.15	**1.51**
**M < 30**	0.04	0.47	0.07	0.59	0.13	0.64
**F 30–50**	0.15	0.54	0.14	0.77	0.11	1.12
**M 30–50**	0.08	0.21	0.18	0.31	0.24	0.32
**F 50–70**	**1.05**	0.26	**1.63**	0.43	**2.02**	0.51
**M 50–70**	0.85	0.14	1.23	0.19	1.62	0.23
**F > 70**	0.89	0.17	1.51	0.30	1.85	0.39
**M > 70**	0.32	0.28	0.38	0.41	1.07	0.46
**TOTAL**	3.53	2.93	5.38	4.25	7.18	5.17

**Table 3:** Evolution of DHDs for antipsychotics by age and gender in the period 2019–2021. PZ = city of Potenza. PROV = municipalities of Avigliano, Barile, Lauria, and Maratea in the province of Potenza. DHD = DDDs for 1000 persons/day. F = female. M = male. Highest value for each age group and year is written in bold.

ANTIPSYCHOTICS						
	DHD 2019		DHD 2020		DHD 2021	
CLOZAPINE	PROV	PZ	PROV	PZ	PROV	PZ
**F < 30**	7.42	4.53	5.72	10.84	9.11	27.31
**M < 30**	6.36	2.27	6.57	7.59	7.84	18.73
**F 30–50**	11.23	3.65	10.17	8.58	13.56	5.42
**M 30–50**	**52.97**	1.97	**84.75**	3.45	**121.20**	2.66
**F 50–70**	30.09	2.56	20.76	6.01	37.50	7.69
**M 50–70**	42.38	3.45	74.16	5.52	95.35	4.14
**F > 70**	9.11	3.94	21.19	8.87	70.77	16.36
**M > 70**	13.77	**28.98**	25.64	**33.81**	76.49	**26.81**
**TOTAL**	173.32	51.36	248.96	84.68	431.82	109.12
	**DHD 2019**		**DHD 2020**		**DHD 2021**	
**QUETIAPINE**	**PROV**	**PZ**	**PROV**	**PZ**	**PROV**	**PZ**
**F < 30**	49.0	**112.8**	65.4	**225.6**	59.9	**330.2**
**M < 30**	16.3	73.5	54.5	148.9	46.3	260.5
**F 30–50**	46.3	121.0	74.9	218.6	81.7	323.2
**M 30–50**	50.4	57.0	69.5	90.6	66.7	135.0
**F 50–70**	54.5	79.8	284.7	186.9	517.6	218.6
**M 50–70**	53.1	78.6	514.9	199.0	309.2	281.4
**F > 70**	**476.7**	70.3	**612.9**	133.1	**1373.0**	197.7
**M > 70**	279.2	39.3	453.6	49.4	696.0	62.7
**TOTAL**	1025.7	632.4	2130.3	1252.2	3150.5	1809.2

**Table 4:** Evolution of DHDs for antidepressants by age and gender in the period 2019–2021. PZ = city of Potenza. PROV = municipalities of Avigliano, Barile, Lauria, and Maratea in the province of Potenza. DHD = DDDs for 1000 persons/day. F = female. M = male. Highest value for each age group and year is written in bold.

ANTIDEPRESSANTS						
	DHD 2019		DHD 2020		DHD 2021	
PAROXETINE	PROV	PZ	PROV	PZ	PROV	PZ
**F < 30**	0.17	0.64	0.16	0.92	0.24	**1.35**
**M < 30**	0.35	0.41	0.56	0.60	0.42	0.90
**F 30–50**	0.15	**0.77**	0.56	0.92	1.14	1.16
**M 30–50**	0.32	0.64	0.31	0.83	0.28	1.07
**F 50–70**	**1.13**	0.61	**1.68**	**1.04**	**1.24**	1.19
**M 50–70**	0.34	0.54	0.35	0.60	0.84	0.68
**F >70**	0.29	0.07	0.76	0.08	1.10	0.07
**M >70**	0.13	0.08	0.06	0.09	0.14	0.09
**TOTAL**	2.88	3.75	4.44	5.07	5.41	6.51
	**DHD 2019**		**DHD 2020**		**DHD 2021**	
**CITALOPRAM**	**PROV**	**PZ**	**PROV**	**PZ**	**PROV**	**PZ**
**F < 30**	0.34	0.03	0.29	0.11	0.37	0.14
**M < 30**	0.31	**0.08**	0.25	**0.16**	0.56	**0.30**
**F 30–50**	0.48	0.03	0.82	0.07	0.50	0.14
**M 30–50**	1.12	0.02	1.17	0.03	1.38	0.04
**F 50–70**	0.28	0.03	0.17	0.05	0.34	0.05
**M 50–70**	**1.37**	0.05	**1.53**	0.06	**1.73**	0.13
**F >70**	0.51	0.07	1.12	0.09	1.27	0.12
**M >70**	0.28	0.06	0.88	0.07	1.16	0.08
**TOTAL**	4.68	0.38	6.23	0.63	7.30	1.00

Clozapine in the city of Potenza has the highest DHD value recorded for men over 70 years old in 2020 (33.81 DHD out of a total of 84.68 DHD for the same year). In the municipalities, the highest DHD value is recorded for men aged 30–50 years in 2021 (121.20 DHD out of a total of 431.82 DHD for the same year). Clozapine shows a significant increase in consumption among women aged 50–70 between 2019 and 2020, as well as among men aged 30–50 between 2020 and 2021 (*p* < 0.05). In the city of Potenza, quetiapine has the highest DHD value recorded for women under 30 years old in 2021 (330.2 DHD out of a total of 1809.2 DHD for the same year). In the municipalities, the highest DHD value is recorded for women over 70 years old in 2021 (1373.0 DHD out of a total of 3150.5 DHD for the same year). Quetiapine shows a significant increase in consumption among men aged 30–50 between 2019 and 2020, as well as among women aged 50–70 between 2020 and 2021 (*p* < 0.05) (Table 3).

Paroxetine in the city of Potenza has the highest DHD value recorded for women under 30 years old in 2021 (1.35 DHD out of a total of 6.51 DHD for the same year). In the municipalities, the highest DHD value is registered for women aged 50–70 years in 2020 (1.68 DHD out of a total of 4.44 DHD for the same year). Paroxetine shows a significant increase in consumption among women aged 50–70 between 2019 and 2021 (*p* < 0.05). In the city of Potenza, citalopram has the highest DHD value recorded for men under 30 years old in 2021 (0.30 DHD out of a total of 1 DHD for the same year). In the municipalities, the highest DHD value is registered for men aged 50–70 years in 2021 (1.73 DHD out of a total of 7.30 DHD for the same year). Citalopram shows a significant increase in consumption among women aged 30–50 between 2020 and 2021, as well as among men under 30 years old between 2019 and 2020 (*p* < 0.05) (Table 4).

## 4. Discussion

This study highlighted the increase in the consumption of anxiolytics, antidepressants, and antipsychotics during the COVID-19 pandemic. Comparing the results obtained from our research, based on temporal trends, starting from 1 January 2019 (the year before the pandemic) until 31 December 2021, we observed an increase in the sales of all the pharmacological categories examined firstly for anxiolytics followed by anti-depressants, and finally for antipsychotics.

Previous studies also considered the effects of COVID-19 on mental health by analyzing the increase in drug consumption.

A multinational study conducted in France, Germany, Italy, Great Britain, South Korea, and the USA highlighted an increase in the consumption of anxiolytics, antidepressants, and antipsychotics during the pandemic period beyond the initial phase of the pandemic [24]. Also in Germany, an increase in the onset of anxiety-related disorders was observed from January to June 2020 compared to the same period in 2019 while, interestingly, the prevalence of use of antidepressants, anxiolytics, and herbal sedatives decreased between 2019 and 2020 [25].

A study conducted by AIFA also confirmed an increase in the consumption of psychotropic drugs, particularly anxiolytics; indeed, in 2020, there was a 12% rise in the use of anxiolytic medications, especially in central regions such as Marche (+68%) and Umbria (+73%) compared to the previous year [26]; this trend was accompanied by an increase in requests for psychological help and support during the lockdown as the Italian Ministry of Health declared more than 50,000 calls to the telephone number for psychological support activated by the Ministry of Health [27].

In our study, the peak consumption occurred in 2021, unlike the multinational study, the German one, and the AIFA report, where the pharmacological increase was observed in the initial phase of the pandemic.

Moreover, referring to the gender and age groups, Santomauro et al. showed how the pandemic led to an increase in depressive and anxiety disorders in Australian females more than males, and younger age groups were more susceptible to both disorders than the older one [28]. A study conducted in France observed an increase in the consumption of anxiolytics, antidepressants, and hypnotics among the age groups 12–18 and 19–25 years. The consumption was higher in women than in men, except for hypnotics, where consumption was similar between genders [29].

Analyzing the DHDs by gender in our study, women’s consumption is higher compared to men, both in provincial towns (52%) and in the regional capital (56%) in according to the previous studies. The reason for this can be attributed to the fact that it was primarily women who bore the brunt of the social impact of the pandemic, particularly in terms of increased work overload and family responsibilities such as childcare and the management of household activities, which made it even more difficult to balance work and private life.

Analyzing the DHDs by age, in the provincial towns, the adults aged between 50 and 70 years and elderly individuals over 70 years old were the people who most frequently used the medications examined. The elderly group appears to be the most affected because, in the small towns of the south, particularly in Basilicata, there is a higher number of elderly people. However, in the city of Potenza, consumption increased among young people under 30 years old. One of the main explanations for the increase is the unprecedented stress caused by the social isolation resulting from the pandemic.

The COVID-19 pandemic not only led to an increase in drug consumption but also had significant repercussions on people’s mental health.

According to the results of the study conducted by the Department of Biomedical Sciences at the Humanitas University, the COVID-19 pandemic had a significant impact on individuals’ psychological and emotional well-being [30]. This study found that between the first and the second wave, 16% and 18.6% of participants, respectively, developed a mental disorder, primarily depressive (8%) and anxiety-related (11%) disorders.

The global prevalence of anxiety and depression increased by a massive 25% in the first pandemic wave [31]. These findings emphasize the relationship between external environmental stress and personal biological vulnerability in the onset of psychotic symptoms [32,33]. Particularly acute and intense stress triggers can, in fact, facilitate the onset of symptoms and the early manifestation of psychiatric disorders even in those without the known risk factors for psychosis.

Overall, the risk of mental illness increased by 60% in people who contracted COVID-19. This means that for every 1000 people, 65 more individuals who had COVID-19 will develop mental disorders or need psychotropic medication each year, compared to those who did not get sick [34].

The increase in the consumption of anxiolytics, antidepressants, and antipsychotics during the COVID-19 pandemic reflects a rise in psychological disorders caused by external factors such as social isolation, fear, and a feeling of uncertainty. Although these medications are essential for short-term treatment, a more comprehensive and preventive approach is needed to address mental health in future crises. COVID-19 pandemic highlighted the weaknesses in psychological support systems and access to effective treatments, suggesting the need for reform in health policies. However, this phenomenon should not only be seen as a reaction to the emergency but also as an opportunity to rethink the management of mental health. To better prepare people to face future similar situations, such as global health crises or emergencies, it is crucial to develop an integrated approach that involves mental health, education, and social resilience. This can be achieved by increasing investments in strategies that promote mental well-being, implementing targeted interventions to prevent conditions like anxiety, depression, and psychiatric disorders, and addressing acute conditions more promptly and effectively.

In fact, globally, there has been a severe lack of resources dedicated to mental health. This is demonstrated by the WHO Atlas Report, which emphasized that in 2020, countries worldwide spent on average just over 2% of their healthcare budget on mental health; many low-income countries reported having fewer than one mental health specialist per 100,000 people, making it urgent to coordinate an intervention to improve access and management of treatments, promoting greater awareness of the risks associated with the indiscriminate use of psychotropic drugs [31,35].

First-level psychological support with rapid patient in-take, use of psychological tools for prevention and early detection of at-risk situations, individual tele-assistance interventions large-scale psychological support services, and the management of home-based psychological care can protect and help, especially those more vulnerable to stress, supporting them in facing future challenges [36]. Strengthening community support networks, alongside integrating psychological support in schools and workplaces, can foster psychological resilience. This approach teaches individuals how to cope with uncertainty and negative situations, even in difficult times. Additionally, investing in alternatives to medication, such as cognitive-behavioral therapy, mindfulness, and other forms of psychotherapy, will better equip us to handle emergency situations.

Prevention must be a priority. Investing in educational programs for both the public and healthcare workers is essential. Training healthcare workers to recognize the first signs of distress, treat psychological disorders, and refer patients to appropriate services when necessary, will help improve the overall response to mental health challenges [37].

Lastly, organizing awareness campaigns about the importance of mindful medication use, guided by professionals and avoiding self-medication during times of stress is critical. Raising awareness about mental health risks and available resources will play a key role in educating people about the responsible use of medications.

Dévora Kestel, Director of the Department of Mental Health at WHO, summarized the situation as follows: “Although the pandemic has generated interest and concern about mental health, it has also revealed a historical underinvestment in mental health services. Countries must act urgently to ensure mental health support is available for everyone.”

### Strengths and Limitations of the Study

Some limitations should be considered for our study. First, the anonymization of the data prevented the identification of drug use at an individual level, making it impossible to assess adherence to them. This is a common limitation of studies based on prescription registers, where it is difficult to determine whether the same population has requested higher doses or if there has been an expansion in the number of individuals receiving treatment [38,39]. Secondly, we were unable to analyze the prescriptions made by private healthcare providers, which could not be quantified because the information was not available so that the real consumption will be higher than that obtained by our study. Lastly, the drugs included in the study are two per category and constitute the most sold drugs. We do not have data on other medications such as other selective serotonin reuptake inhibitors (SSRIs) or other benzodiazepines.

The study’s strength lies in the fact that we have clean data on the consumption of the drugs examined, and by collecting the data directly from the pharmacies, in the most representative cities, we have the actual and effective pharmacological consumption data. The availability of data from 2019 to 2021 for comparisons also strengthens the study, along with the DHD values for each drug, which show how there has been an increase in the population treated during the study period.

## 5. Conclusions

The COVID-19 pandemic created an entirely new crisis: the psychological crisis alongside the health crisis, because of feelings of uncertainty, fear, and loneliness due to containment measures, such as lockdowns, school closures, office shutdowns, and business restrictions. All these factors contributed to an increase in stressful and anxiety-inducing triggers, leading to a rise in stress, anxiety, and depression, which resulted in an increase in the prescription and consumption of anxiolytics, antidepressants, and antipsychotics.

Our study, like others, highlighted the rising trend in the consumption of psychotropic medications during the COVID-19 pandemic, particularly in middle-aged women.

Awareness campaigns and large-scale psychological support services can protect and help especially those more vulnerable to stress, supporting them in facing future challenges. Future research should investigate the potential causes behind these trends and assess whether policy changes, medical guidelines, or broader health interventions are contributing to improve the population’s mental health, limiting the increased use of psychotropic medications.

## Figures and Tables

**Table 1:** Evolution of the consumption by pharmacological subgroups in the period 2019–2021 in the city of Potenza and in the municipalities of Avigliano, Barile, Lauria, and Maratea. ∆ = annual percentage variation. % = percentage of consumption in the population. n = total of boxes sold.

PHARMACOLOGICAL SUBGROUP IN POTENZA CITY	2019 (%)	2020 (%)	2021 (%)	TOTAL (n)	∆ 2020/2019 (%)	∆ 2021/2019 (%)
ANXIOLYTICS (N05B)	23.16	33.17	43.67	26,023	43.2	88.5
ANTIPSYCHOTICS (N05A)	18.27	34.09	47.64	8316	86.6	160.8
ANTIDEPRESSANTS (N06A)	23.81	32.89	43.30	14,649	38.1	81.9
**PHARMACOLOGICAL SUBGROUP IN MUNICIPALITIES**	**2019** **(%)**	**2020** **(%)**	**2021** **(%)**	**TOTAL** **(n)**	**∆ 2020/2019 (%)**	**∆ 2021/2019 (%)**
ANXIOLYTICS (N05B)	19.12	33.62	47.26	15,564	75.8	40.6
ANTIPSYCHOTICS (N05A)	18.14	31.62	50.24	8661	74.3	58.9
ANTIDEPRESSANTS (N06A)	24.42	34.48	41.10	12,169	41.2	19.2

## Data Availability

All data generated or analyzed during this study are included in this published article.

## Supplementary Materials

The following supporting information can be downloaded at https://www.mdpi.com/article/10.3390/life15040652/s1. Supplementary Table S1: Drug prescription frequencies analysis.
