# Supplementary material for: Impact of COVID-19 Pandemic on Consumption of Anxiolytics, Antipsychotics, and Antidepressants in South Italian Region
Source: Life (Basel). 2025 Apr 16;15(4):652. doi: 10.3390/life15040652 (PMC12028633; doi:10.3390/life15040652)
Supplement: Supplementary file 1 [file life-15-00652-s001.zip › life-3513252-supplementary.pdf]

Drugs Prescription Frequencies Analysis

Table S1. This table summarizes the absolute and relative frequencies of drug prescriptions analyzed in the years 2019, 2020 and 2021, divided between the province and city of Potenza.

DRUGS	YEAR	Absolute Frequency Province	Relative Frequency Province	Absolute Frequency Potenza	Relative Frequency Potenza
**ALPRAZOLAM**	2019	3892	0.0829	6928	0.1475
	2020	5928	0.1262	10050	0.2140
	2021	7912	0.1685	12234	0.2606
**PAROXETINE**	2019	2264	0.0630	6332	0.1763
	2020	3492	0.0972	8566	0.2385
	2021	4258	0.1185	11000	0.3063
**QUETIAPINE**	2019	1506	0.0719	1996	0.0954
	2020	3128	0.1495	3952	0.1889
	2021	4626	0.2211	5710	0.2729
**CLOZAPINE**	2019	1636	0.1254	1042	0.0799
	2020	2350	0.1802	1718	0.1317
	2021	4076	0.3126	2214	0.1698
**CITALOPRAM**	2019	3680	0.2076	644	0.0363
	2020	4900	0.2764	1070	0.0603
	2021	5744	0.3240	1686	0.0951
**LORAZEPAM**	2019	2060	0.0568	5128	0.1415
	2020	4536	0.1252	7212	0.1990
	2021	6800	0.1876	10494	0.2896
